# Medicine Maker: An Outreach Activity for Pharmaceutical Manufacturing and Health Literacy

**DOI:** 10.1021/acs.jchemed.1c00915

**Published:** 2022-02-24

**Authors:** Martin McHugh, Sarah Hayes, Lidia Tajber, Laurie Ryan

**Affiliations:** † SSPC, the SFI Research Centre for Pharmaceuticals, Bernal Institute, University of Limerick, Limerick V94 T9PX, Ireland; ‡ Department of Sport and Health Sciences, Athlone Institute of Technology, Athlone N37 HD68, Ireland; § The School of Pharmacy and Pharmaceutical Sciences, Trinity College Dublin, Dublin D02 PN40, Ireland

**Keywords:** Public Understanding/Outreach, General Public, Inquiry-Based/Discovery Learning, Drugs/Pharmaceuticals

## Abstract

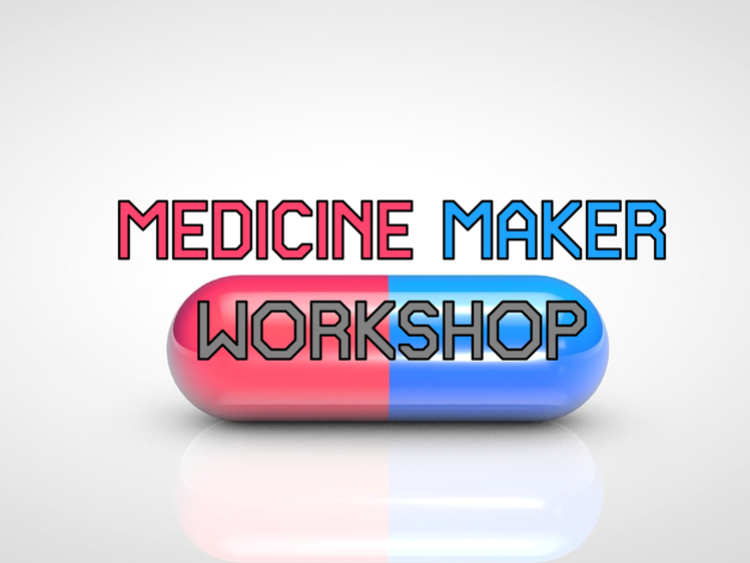

Public engagement in medicine has become more important in promoting population health management and literacy. Medicine is a topic of great societal importance, and many public engagement activities have been developed to promote this area. However, they often narrowly focus on patient groups, diseases, a singular pharmaceutical drug or analytical technique. Despite the importance of these activities, general audiences are still heavily reliant on doctors and pharmacists for information about their medicine and lack basic knowledge around medication use and personal safety. Given this, a broader engagement approach is warranted to target health literacy among the wider public. “Medicine Maker” is a hands-on public engagement workshop that provides audiences with the opportunity to “manufacture” and inspect the quality of proxy or “dummy” medicine through guided inquiry. Here, we detail the development of the Medicine Maker workshop from its origins in the teaching of Irish third-level pharmacy students, to its initial application with a variety of lay audiences. Formal and informal feedback from participants indicates that the workshop can help foster a more critical understanding of medicine manufacturing, quality control, and personal health.

## Introduction

Heath literacy refers to a person’s ability to understand, evaluate, and engage with personal health information.^1−3^ In line with literacy, health literacy comprises a set of skills that vary among populations and refers to one’s ability to engage with healthcare professionals and navigate healthcare institutions and use of medicinal products.^2^ Several factors influence health literacy including age, socioeconomic status, education, and disability.^4^ Results from the 2003 National Assessment of Adult Literacy (NAAL) indicate that only 12% of American adults have high levels of health literacy.^5^ More recent results from Ireland demonstrate that 21.3% of people have “excellent” health literacy, 38.7% have “sufficient” health literacy while 40% have “problematic” or “inadequate” levels of health literacy.^6^

In a broader attempt to enhance awareness and scientific literacy among the Irish public, Science Foundation Ireland (SFI) (Irish equivalent of National Science Foundation) funds 16 research centers, all of which have a public engagement mandate. There is now an embedded expectation among research funders pertaining to public engagement and its impactful facilitation in third level institutions.^7^ SFI have strongly promoted this agenda since 2013. As one of the 16 centers, SSPC (Research Centre for Pharmaceuticals) has been designing and implementing public engagement events spanning from digital campaigns to school workshops.

As noted, public knowledge of medicine and personal health can be limited. Moreover, medicine is a topic that resides at the intersection between science and society.^8^ Given the status quo, public engagement is pivotal to an informed and health literate population.^9^ Numerous authors argue similar positions calling for inclusive public engagement that encourages bidirectional discussion around these issues.^6−8,10^

While there is consensus around the need for public engagement, outreach providers tend to focus on specific subject areas or groups such as regenerative^11^ or geriatric medicine.^7^ Broader efforts such as “Pharmacists in Schools”^12^ and work in the area of antibiotic resistance^13^ are promising initiatives and Medicine Maker aims to build on this by providing detailed insights into the design of implementation of engagement activities in this space. Additionally, there is a public engagement gap covering the basics of how medicines are made, drug safety and quality control, access to information, and pharmacovigilance. The former reflects the core aspects of health literacy this workshop aims to address while also being open to ancillary areas of health literacy brought forward by audience interactions. This is facilitated through experiential hands-on public engagement where medicine perceptions and misconceptions can be discussed while also providing a stimulating and informative introduction to the topic.^7,14^

## Concept and Educational Objectives

The core educational objective of the Medicine Maker workshop is to promote health literacy through hands on and active engagement. In achieving this, we ask participants to use and operate a capsule filling plate to make dummy capsules as a way to parlay into discussions around pharmaceutical manufacturing, quality control of medicines, and pharmacovigilance. The inception of the idea arose from a collaboration between the SSPC public engagement team and an academic partner who uses a capsule filler to teach university pharmacy students. With pedagogical modification, the same equipment can be used with nonspecialist audiences. From a teaching and learning design perspective, we adopted a guided inquiry approach so that participants would feel comfortable partaking in the workshop. Research indicates that guided inquiry, as an instructional method, augments student engagement.^15^ From our experience (as former secondary school teachers (high school equivalent)), we have found that many formal educational approaches can be readily adapted to informal contexts. These approaches often begin with a question or challenge and are followed by a practical investigation.^16^ In the case of Medicine Maker, the challenge is making dummy capsules that will pass simple quality control tests. The purpose of introducing the topic of quality control is to support discussion around how pharmaceutical companies ensure that all medicine they produce have the same composition. With any medicine, the end user places a high level of trust in the manufacturer. A key objective of the workshop is that audiences recognize the important connection between the manufacturer, broader society, and regulatory bodies.

Finally, there was a requirement for the workshop to be adaptable to a variety of contexts and audiences. For example, when working with active retirement groups, an instructor can demonstrate the equipment and give the participants the choice of actively taking part given that some members may have issues with dexterity. When working in a school environment, the guided inquiry approach can be fully adopted due to the learning setting. Inherent to informal education is diversity in terms of context from hotels to libraries and classrooms. An additional consideration is that equipment needs to be easily transported, cleaned and refilled. This model of bringing “easy to implement”^17^ public engagement from a university lab to a public space is well established. This model was pioneered in Ireland and the United Kingdom with the Spectroscopy in a Suitcase Programme from the Royal Society of Chemistry.^18^

Such design considerations underline the need for trial runs and testing. To pilot the workshop, we worked in a variety of settings with a diversity of audiences. The range of environments acts as a stress test for the workshop and allows the discovery of design kinks or flaws. Once complete, a formal evaluative effort was implemented with secondary school students in a classroom setting. Within the literature, it is argued that there is a meaningful connection between levels of health literacy among adolescents and positive health behaviors.^1^ The appropriate use of medicine is a life skill and one that students can carry forward as they transition into adulthood.^19^ Given the above, we endeavored to evaluate the impact of the workshop on an important target group.

## Medicine Maker

**Figure 1:**
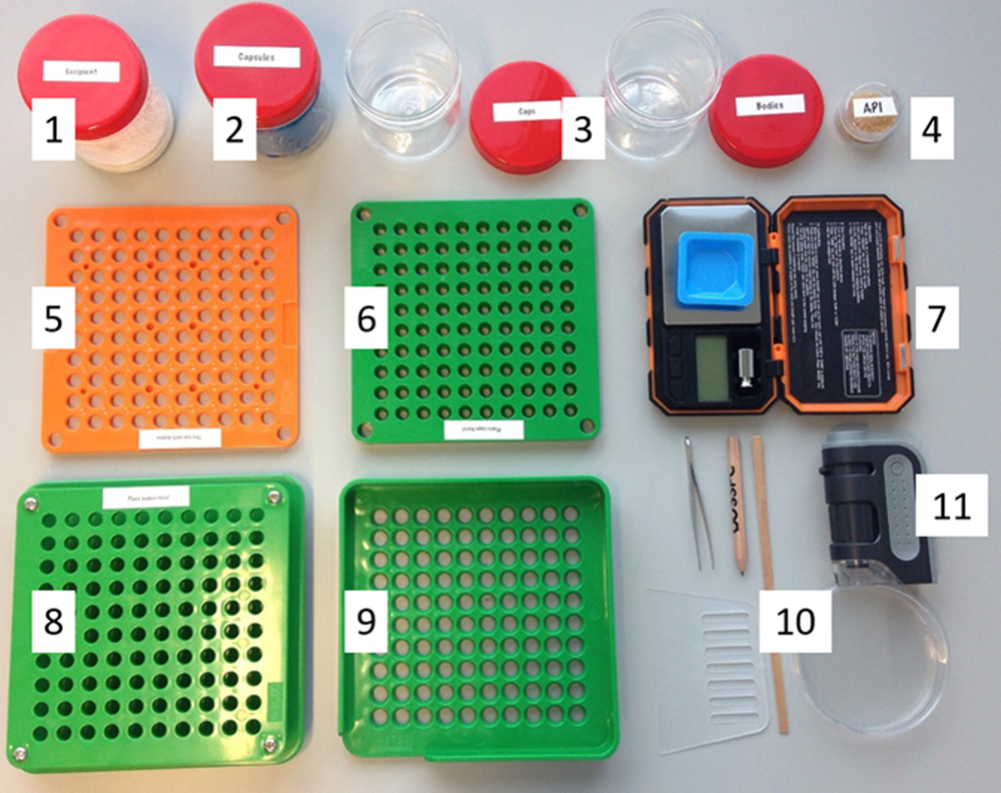
Representative materials within each medicine maker kit: (1) flour, (2) capsules, (3) containers for separating capsules into caps and bodies, (4) brown sugar, (5) middle plate, (6) cap plate, (7) digital milligram scales, (8) body plate, (9) sorting plate, (10) scraper, Petri dish, tweezers, and coffee stirrer, and (11) Carson pocket microscope.

In July of 2019, we applied for the SFI Science Week Funding Call. Science week is a national campaign that takes place during November of each year, and the call for funding is open to small scale events as well as large scale festivals. The total funding awarded for the workshop was €2650, and these funds were used to cover the cost of equipment and travel to audiences/participants throughout the week. A complete list of equipment that is contained in a medicine maker box can be seen in Figure 1. A breakdown of equipment costs is listed in Table 1 below.

**Table 1:** List of Equipment and Costs to Create 16 Medicine Maker Kits

equipment	cost	reusable (Y/N)
flour 5 kg (used for multiple workshops)	€6	N
capsule filler plates × 16	€450	Y
empty gelatin capsules (500 pack) × 150	€759	N
Carson travel microscope × 16	€300	Y
4 L storage boxes × 16	€94	Y
label maker	€81	Y
milligram scales × 16	€287	Y
weight boats (1000 pack)	€38	N
sugar 5 kg	€5	N
250 mL round storage containers × 98	€91	Y
all other equipment (Petri dishes etc.) sourced from university laboratories		

The equipment is designed to neatly fit inside a 4 L clear container, and each box represents a single kit. A total of 16 kits were compiled, meaning that the maximum number of participants would be 45 (1 kit between 3 participants) while the optimal would be 30 (1 kit between 2 participants) or less. A spare kit was developed for the instructor to demonstrate. Given that the kits required transportation to and from various sites, they were designed with volume in mind and they can readily fit in a small sized car. Moreover, the kits are stackable to support their ease of transportation.

The core of the kit is the capsule filling plate, and the majority of other equipment is designed to support its use. It should be noted that the plates come in standard sizes depending on the size of the gelatin capsule to be filled. We decided to use size 00 (750 mg capacity) capsules and matching plates as these capsules are large enough to be easily manipulated and are common among medicines and supplements. Capsules were sourced from an online retailer, and 1500 are used in every workshop (100 per kit). This is the largest continual expense in running the workshop, and as such we are engaging with industry sponsors who manufacture capsules to cosponsor the workshop delivery. Another potential obstacle is having to count out 1500 capsules per workshop. We worked out that 250 mL containers will hold approximately 110 capsules, allowing them to be “poured” into the containers until filled. Calculations like this are time-savers when preparing, cleaning and refilling kits. Other design elements include the use of flour and brown sugar as the dummy excipient and Active Pharmaceutical Ingredient (API), respectively. These compounds were selected given that many medicinal products comprise crystalline APIs and excipients, which are often amorphous in nature and white in color. They were also selected in case any participants ingested the gelatin capsules or other components. In addition, this allows the participant to readily distinguish between the two based on color and structure. These differences are more readily visible when using the Carson microscope (Carson MicroBrite Plus).

## Workshop Delivery

**Figure 2:**
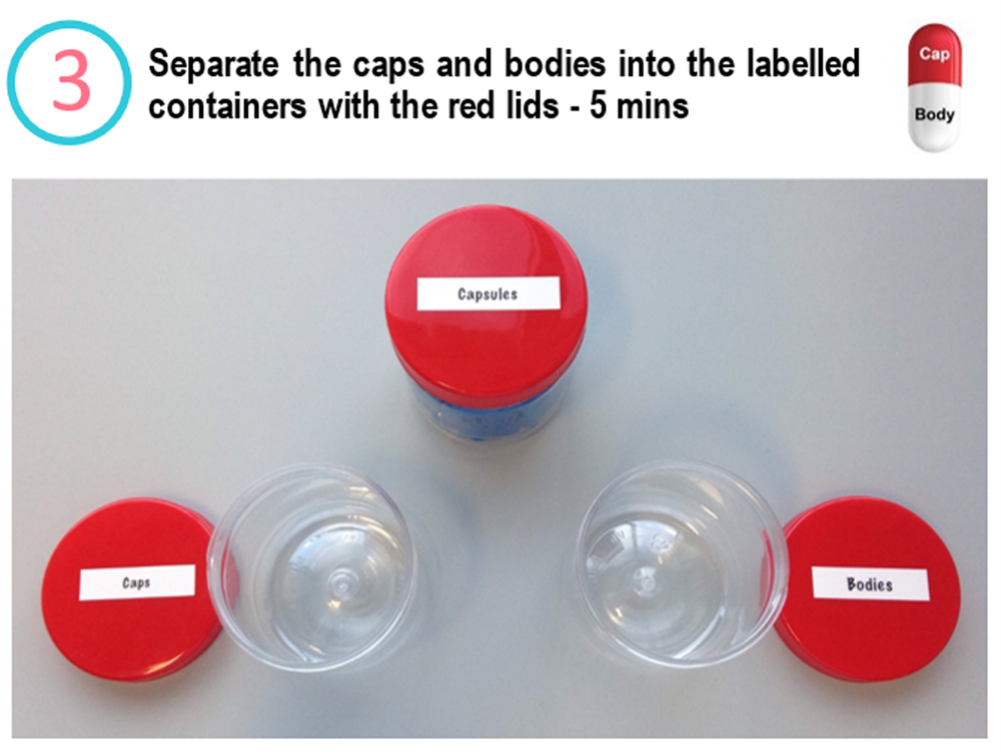
Slide from the Medicine Maker workshop. Step number 3 in which participants are asked to separate their capsules into caps and bodies.

Pertaining to delivery of the workshop, the main resource for the instructor is the PowerPoint presentation. This consists of a brief introduction to the pharma industry in Ireland. This is immediately followed by the goal of the workshop, quality control. Participants are presented with the following challenge on a PowerPoint slide: “To make medicine that is perfectly formed and exactly the same using a medicine maker kit”. They are also then informed that they will be helped through a step-by-step process. All participants are asked to lay out their equipment in a similar fashion to Figure 1. Participants are asked to describe different types of medicine, and this questioning is used as a way of introducing the capsules. The morphology of capsules is explained and demonstrated along with an explanation of APIs and excipients, their roles in medicinal manufacture and in the body. Participants are also told about the importance of accuracy and that their “medicine” will be tested to see if it “passes” or “fails” some simple quality control tests. Moving forward, participants follow the guided steps of dividing the capsules into caps and bodies (Figure 2), setting up the pill plate and filling half of their pill plate with the capsule bodies. These steps are demonstrated to participants while instructors can also visit individual groups who may need help. While each step has an associated time frame, this is a loose guide and instructors can readily vary the times to facilitate a diversity of groups. This is the same for the overall duration of the workshop which is typically 80 min.

**Figure 3:**
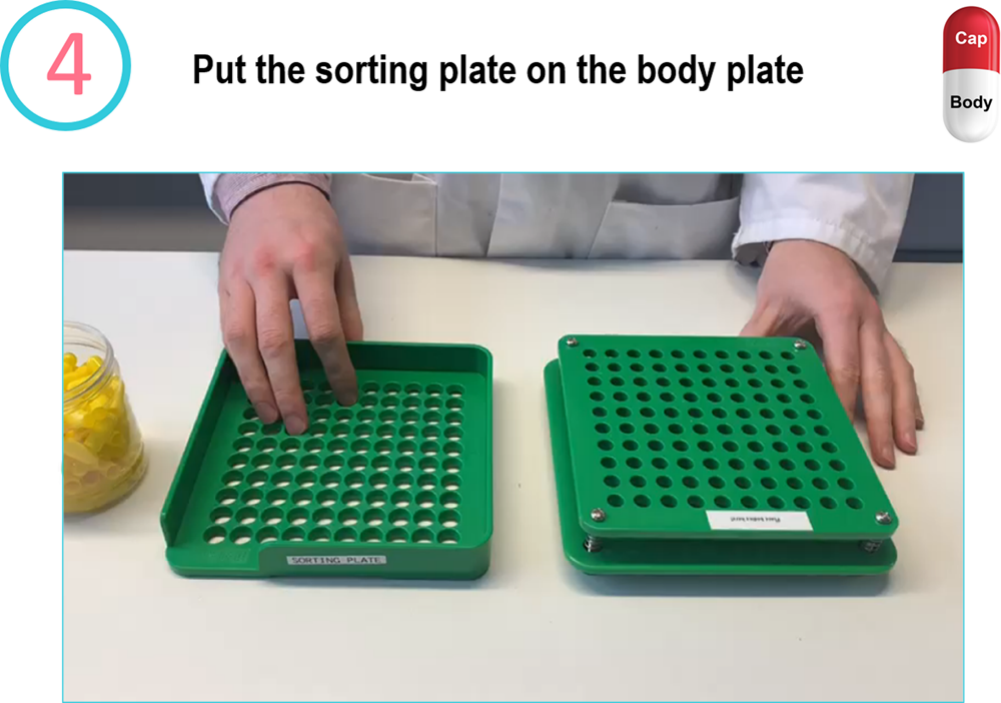
Slide from the Medicine Maker workshop. Step number 5 in which participants are asked to fill their capsule bodies in their capsule plate. Note that they are asked to conduct this step inside the lid of the medicine maker box given that this step can get quite messy.

At this point, participants reach a critical juncture, filling the capsules. Participants are presented with the slide in Figure 3 and asked to fill the dummy capsules with both their excipient and API. The goal of quality control is reinforced and that they are allowed use any method they want to complete the task.

This is the core inquiry aspect of the workshop and individual groups are encouraged to figure out a process for completion of the task. They are given approximately 20 min and are again informed that all of their capsules must be the compositionally identical pertaining to API and excipient. They must also keep the weight and visual neatness (also referred to as elegance) of their capsules in mind. To cater for academic diversity, this time is used by instructors to move around the room and talk to all groups and give some guidance if required. Once complete, a stepwise approach is again adopted so that participants can attach the caps to the bodies of their capsules. All filled capsules are then transferred into a container ready for inspection. Following this, participants are asked if they think their capsules are equal and if they would pass a quality control test. This promotes the participants thinking about the difficulty in making sure that tablets or capsules are all the same. Simple and relevant examples such as crushing or splitting tablets are used to highlight the importance of making medicine that is compositionally balanced. Moreover, participants are also asked if they could tell the difference between their dummy capsules and capsules you can buy from a shop. All of these questions encourage critical thinking from the audience and are used to elicit a brief discussion among the group.

With these questions in mind, participants visually assess their capsules for any defects and break open some of their capsules into a Petri dish to see (with and without microscopes) if they contain both API and excipient. Groups are given a pass or fail at this step, but all groups perform the last step which is weighing the capsules. All groups are asked to take 10 random capsules and weigh them all individually on the digital milligram scales provided. Once recorded, they note the average and then work out if their capsules are within 10% of the average. If all capsules are within the 10% weight variation, they pass; if not, they fail.

This marks the end of the hands-on aspect of the workshop. The presentation continues with a quick overview of high-performance liquid chromatography (HPLC) and spectroscopy as higher-level methods of quality control in industry. Participants are also briefly informed about prescriptions, labeling of medicine, and pharmacovigilance, allowing them to utilize their own prior knowledge and new knowledge from the workshop to discuss medicines and related topics. They are also informed about where they can get more information and asked to tidy up their kits. This marks the end of the workshop. It should also be noted that the workshop is open for questions and discussion at any stage.

### Health and Safety

Medicine Maker has some minor risks. These include irritation to eyes from the sugar and flour, along with the potential for ingestion, especially among younger participants. To avoid both concerns, participants are informed of health and safety risks before the workshop and the Medicine Maker instructor carries saline eye wash at all times.

## Pilot

The Medicine Maker workshop has been implemented a total of 10 times since its inception by two instructors who have delivered the workshop both individually and collaboratively. The initial five workshops occurred during Science Week 2019 and the remainder occurred later on an ad hoc basis (Table 2).

**Table 2:** Interventions for the Pilot and Main Phase of the Study Including Evaluation Methods

pilot	5 workshops (primary school, secondary school, youth group, active retirement group, teacher group)	evaluation during the pilot consisted of reflection, peer discussion, and observation
main phase	5 workshops (4 secondary schools, 1 primary school)	evaluation during the main phase expanded on the pilot through the use of exit card surveys

**Figure 4:**
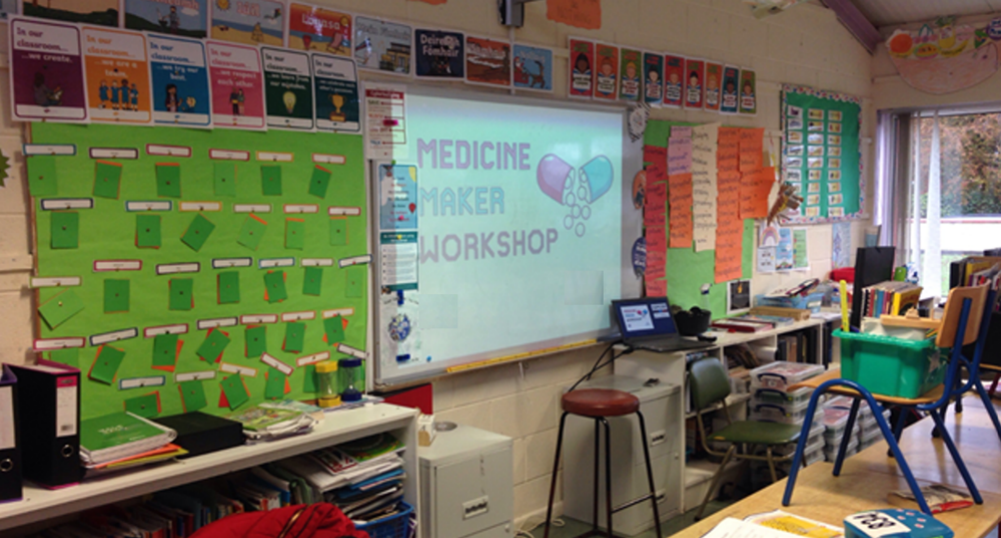
Medicine Maker presentation on an interactive whiteboard in a primary school during Science Week 2019.

The first five workshops were used as a pilot to optimize the design of Medicine Maker. Informal evaluative methods such as peer reflection and discussion along with taking notes of interactions and feedback from participants were all used as ways of carving the workshop into its most effective state. In addition, throughout Science Week, we worked in a diverse array of contexts including a primary school (Figure 4), secondary school, youth group, active retirement group and group of teachers. Although Medicine Maker is primarily designed with schools in mind, the advantage of working in varied contexts is that is “stretches” the workshop and enables the user to actively find areas in which the workshop is under stress. This allows for active corrections and optimization of anything from slides to pedagogy or preparation. This initial evaluative step is essentially internal and examines the workshop protocol rather than directly seeking the views of or impact upon participants. The findings from these five preliminary workshops are outlined below.

During the pilot evaluation stage, we found that the original workshop design worked well and could be readily adapted to most contexts. This included having groups of different sizes and teaching at a variety of paces depending on the age profile. For example, with the youth groups, due to the low numbers present (12), each participant could use a kit individually. Due to the layout of the room, some participants worked on the floor, while others used a pool table as a makeshift desk. However, there were some more bespoke learning outcomes from the pilot.

**Figure 5:**
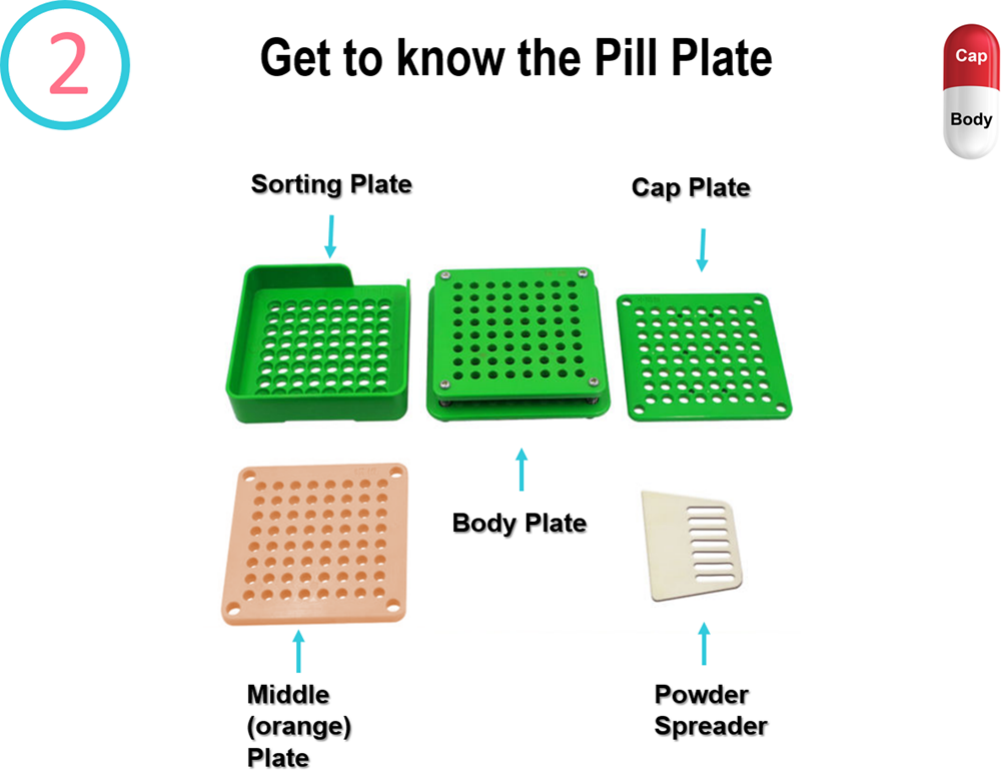
Slide from the Medicine Maker workshop slide deck with the capsule morphology displayed in the top right-hand corner.

First, participants found some of the equipment exciting but also a little intimidating and needed to be reassured that the workshop was straightforward and they would receive help along the way. This is one of the reasons why an equipment slide was added to the presentation so instructors would make an explicit introduction to the capsule filling plate and its components. Some of the younger groups also had to be guaranteed that capsules and equipment were the same across all kits even though there are color variations. Throughout all tasks, we intentionally ensured that all groups maintained the same pace by helping where needed. Participants found the stepwise approach beneficial especially when given clear tasks such as separating capsules into caps and bodies. Indeed, we found that it was advantageous to have the morphology of the capsule on every slide (Figure 5) to support participants throughout all tasks.

**Figure 6:**
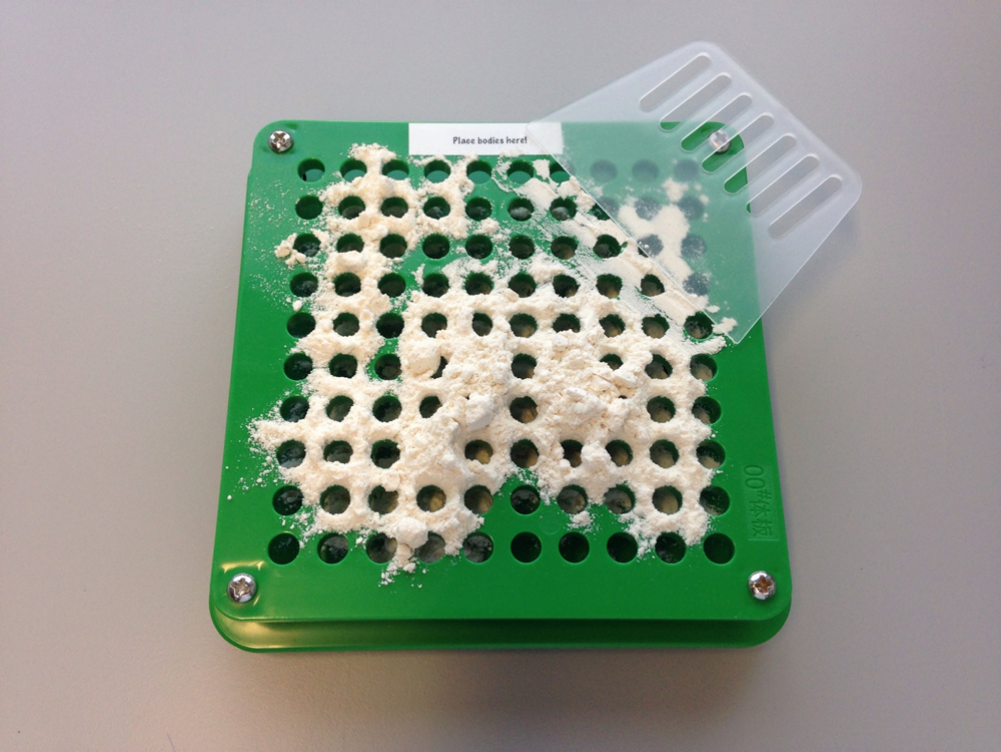
Shows the initial attempt by a participant at spreading their excipient. Their aim is to now use the scraper to evenly spread the flour across all capsules bodies that reside in the holes.

The initial guided steps were excellent at building up the confidence of the participants with the equipment, and this worked favorably when moving onto the inquiry section. Participants are asked to “fill their capsules” utilizing any method they deemed fit as long as all capsules were the same. This brought about a mixture of results with some participants methodically weighing their API and excipient and filling all capsules by hand. Others filled all their capsules with excipient and then added the API, some mixed their API and excipient in a container, others made makeshift tools such as funnels from paper or just simply poured their materials over the capsule filling plate and scraped everything into place (Figure 6).

Furthermore, some groups felt that they had to use all of their API and excipient while others used tweezers to place a single brown sugar crystal into every capsule. Throughout the inquiry process, participants continually asked about the “correct” method to fill the capsules. There is no one technique to filling the capsules, but once the activity was over, the group generated methods were contrasted against each other with an inverse correlation between speed and accuracy. In addition to this, there was an obvious delineation between the neat and messy groups with some spilling flour and sugar on themselves and their workspace. Given this, we made the adjustment that when filling the capsules that they needed to use the lid of the kit box as a base to help ensure cleanliness. Depending on the context, lab coats or aprons can also be used. Despite the variations among the inquiry aspect of the workshop, to the participants surprise, all the complete capsules looked identical. Participants noted how real their dummy medicine looked and felt. They expressed a satisfaction at the work they had completed and looked forward to not only the results of their own quality control tests, but the tests of other groups. This represented a key juncture in the workshop as we found that participants were more open to questions and discussion after this point.

Other issues arose during the quality control part of the workshop. Some participants struggled to use the digital scales and required help, while others struggled with the calculation to ascertain the average weights and the 10% range. This finding is in alignment with the broader literature on student difficulty with calculations.^20^ To compensate, instructors spent more time on this task and maintained a keen awareness that participants may need help with this particular section of the workshop.

Apart from this, participants enjoyed the pass/fail aspect of the activity and took joy in other groups failing the quality control step. Indeed, these aspects of the workshop allowed for a jovial and engaged atmosphere at times. This type of environment supported questions and discussion around real medicine, pharmacies, and pharmaceutical manufacturing. Participants also became acutely aware and verbalized of the potential for counterfeit medicine given the ease at which they had produced 100 dummy capsules per group. This greatly aligned with the health literacy goals of the workshop and the interactions were deemed a success.

After the conclusion of the pilot, the workshop was refined with a more established best practice. It is at this point that we added a formal evaluation in order to get the external or participant perspective. Results from the remaining five workshops conducted are elucidated below.

## Results and Discussion

### Medicine Maker Participant Feedback

**Figure 7:**
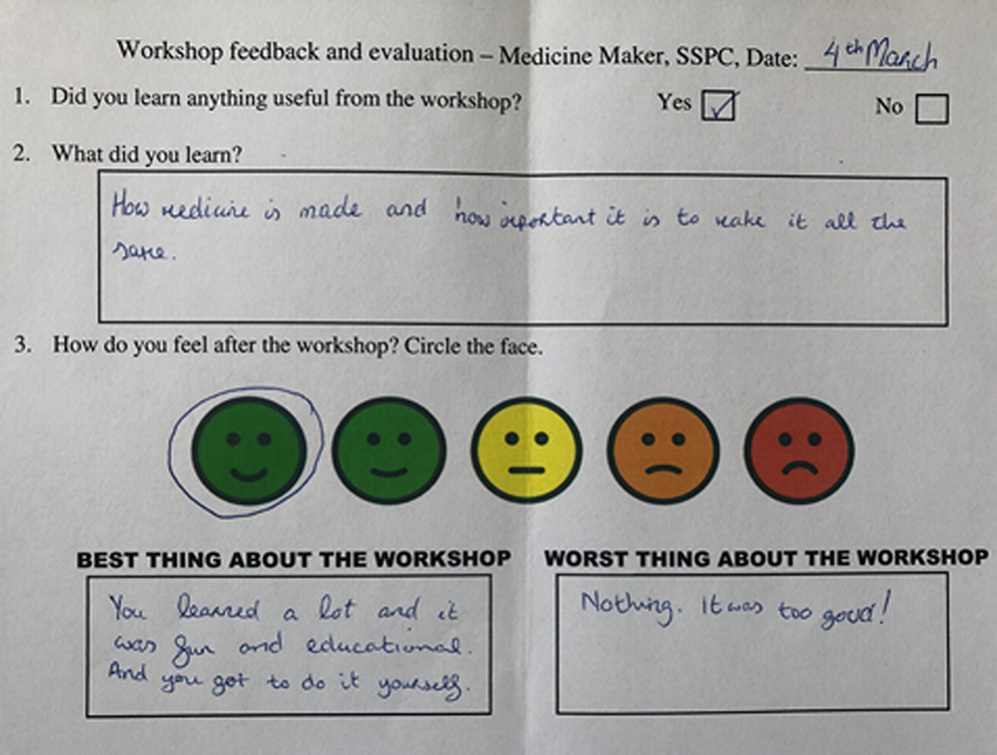
Image of a completed exit card by a student.

Following Science Week, the revised Medicine Maker workshop has been implemented in five different schools with 91 participants (*N* = 91) in what we term the “main phase” building off the pilot. Four of the five were secondary schools with a single primary school. There was a mix of rural and urban schools and students had an age profile from 11 to 16. In order to more formally evaluate Medicine Maker, we employed exit cards (also referred to as exit tickets) post workshop as a quick way of attaining the students’ insights. Exit cards are short deductive surveys commonly used in classrooms.^21^ They are ideal for evaluating the opinions of students given that they are easy to comprehend and answer.^22^ Moreover, they are an unobtrusive and safe way for students to voice their opinion anonymously.^23^ An example of the exit card used throughout the evaluation of Medicine Maker can be seen in Figure 7 below.

As can be observed, the exit card contains two quantitative measures and three qualitative measures. The quantitative measures ask students “Did you learn anything useful from the workshop”, and 99% ticked “Yes”. Additionally, we ask participants to circle the face^24^ that indicated how they felt after the workshop. This was linked to a scale from 1–5 (5 being the most positive). The average score for the workshop was 4.9. These results are positive and typical from students who engage with informal science activities. The benefit of the quantitative measure is that it gives a fast indicator of overall workshop quality and if large modifications are required to any given intervention.

This highlights the importance of a mixed approach in terms of evaluation. The thematic qualitative data is listed in Tables 3–5 below. Students were asked three qualitative questions:

- What did you learn?
- Best thing about the workshop
- Worst thing about the workshop

Pertaining to question 1, students gave a variety of responses. The four broad themes their answers aligned with include learning about the importance of quality control, the composition of medicine, how medicines are made, and to only take prescribed medication from a doctor. The answers indicate the potential of the workshop to promote basic health literacy, particularly given that much of the information taught was seemingly new to the students. One student commented, “even though a tablet may look real, it does not mean it’s safe”, while another noted “pills are made very easily and that if you buy from someone who is not a doctor, it could be filled with anything.” The data also indicated that students took onboard the personal health safety messages embedded within the workshop. Moreover, Table 3 suggests that students had no single dominant take home message, the workshop provided for multiple learning points.

**Table 3:** Results to the Question “What Did You Learn?” (*N* = 91)

importance of quality control	composition of tablets and capsules	only take prescribed medicine	how medicine is made	dangers of buying medicine online	drug safety	size of pharma industry in ireland	how to use a microscope	unanswered
20 (21.99%)	20 (21.99%)	19 (20.89%)	14 (15.39%)	7 (7.71%)	4 (4.37%)	2 (2.19%)	1 (1.09%)	4 (4.37%)

When asked about the “best thing about the workshop”, students indicated that the hands-on inquiry aspects of the workshop were fun and enjoyable (Table 4). Many students noted that “making the capsules” was the best part of the workshop. A key word that continually emerged was “satisfaction”. Students enjoyed how all the equipment aligned to form their final product. As discussed previously, the hands-on aspect of the workshop was fundamental to the inquiry design and inherent in the name of the workshop. There is a strong case that the “maker” facet of the workshop is key to its success particularly with regard to making cautionary topics accessible and interesting.

**Table 4:** Results to the Question “Best Thing about the Workshop” (*N* = 91)

hands-on learning	learning new information	it was fun	it was interesting	the instructors	using the microscope	new experience	break from school	unanswered
49 (53.98%)	11 (12.12%)	8 (8.79%)	6 (6.59%)	5 (5.49%)	5 (5.49%)	2 (2.19%)	1 (1.09%)	4 (4.37%)

Finally, students were asked about “worst thing about the workshop”. The majority of responses indicated that “nothing” was wrong with the workshop, whereas other responses proved more valuable and aligned with the experiences of the instructors. Three categories are presented in Table 5 and are closely interlinked, namely, “need for precision”, “not enough time”, and “too challenging”. All of these categories relate directly to the inquiry section in which students are asked to fill the capsules without instructions and only the guidance that they must be the same and contain both excipient and API. As noted, throughout this section, there was an inverse relationship between speed and accuracy, and this is the entire point of the activity. As one student said, “it was very hard to get the tablets equal.” The fact that some students found the task difficult directly aligns with the goal of the inquiry section. The lesson being that making real medicine is difficult and requires stringent standards. Moving forward, this may need to be made more explicit to participants after the inquiry section of the workshop.

**Table 5:** Results to the Question “Worst Thing about the Workshop” (*N* = 91)

nothing	need for precision	not enough time	too challenging	messy	information learned was scary	unanswered
54 (59.39%)	12 (13.18%)	9 (9.89%)	6 (6.59%)	5 (5.49%)	1 (1.09%)	4 (4.37%)

On the basis of the feedback as a whole, we believe that Medicine Maker provides a good model for teaching and learning about medicine. Although the workshop provides a cautionary tale pertaining to medicinal manufacture and personal health, the guided inquiry approach makes the topic more accessible for a wide range of audiences.

## Conclusion

Medicine Maker is an informal hands-on workshop that can be used in a variety of learning contexts but is particularly suited to schools. The workshop is an effective tool at engaging audiences with the topic of medicine and has the potential to impact areas of participants’ health literacy. By aligning the workshop with the principles of guided inquiry, specific subject areas such as pharmaceutical manufacturing and quality control are made more tangible. This is further bolstered by the introduction of real-world examples around prescriptions and the dangers of buying medicine online. We also outline the benefits of practical design considerations when creating a workshop for use in multiple contexts requiring transit. While the SSPC will continue to run the workshop, we are currently pursuing efforts to further develop the activity with an industry partner. Using a Gradual Release of Responsibility (GRR) model, we can train industry staff to deliver the workshop to classrooms and other settings of interest. This may also allow for the inclusion of HPLC or spectroscopy as additional elements to the current workshop or as a follow up workshop. Furthermore, we may look to include a pre/post-research-based assessment of health literacy to more fully explore the impact of Medicine Maker on participants.

## Supporting Information Available

Slide presentation used with the Medicine Maker Workshop (PDF)
