# Supplementary material for: Medicine Maker: An Outreach Activity for Pharmaceutical Manufacturing and Health Literacy
Source: J Chem Educ. 2022 Feb 24;99(3):1231–7. doi: 10.1021/acs.jchemed.1c00915 (PMC8908739; doi:10.1021/acs.jchemed.1c00915)

Supporting Information

Medicine Maker: An Outreach Activity for Pharmaceutical Manufacturing and Health Literacy

Martin McHugh[1], Sarah Hayes[1], Laurie Ryan[2] and Lidia Tajber[3]

[1]SSPC, the SFI Centre for Pharmaceuticals, Bernal Institute, University of Limerick, V94 T9PX, Ireland

[2]Department of Sport and Health Sciences, Athlone Institute of Technology, N37 HD68, Ireland

[3]The School of Pharmacy and Pharmaceutical Sciences, Trinity College Dublin, D02 PN40, Ireland

Corresponding Author: martin.mchugh@ul.ie

MEDICINE MAKER WORKSHOP

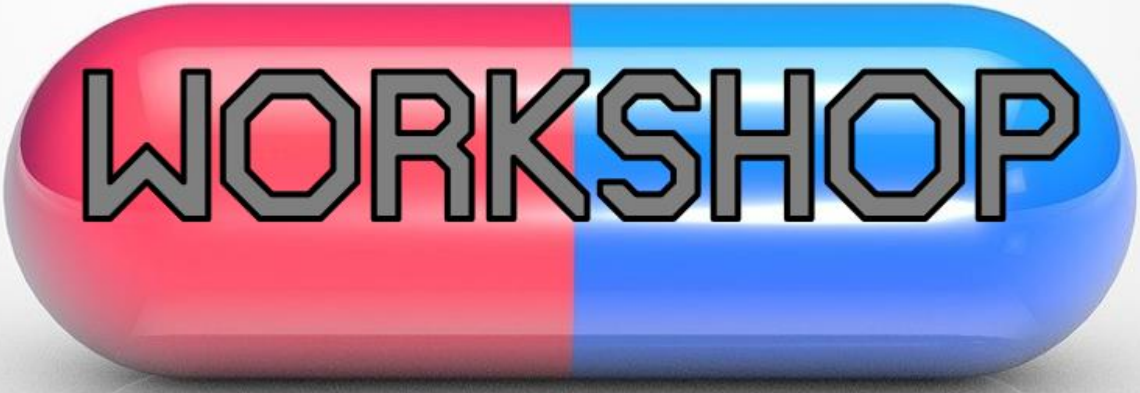

THE GOAL – QUALITY CONTROL

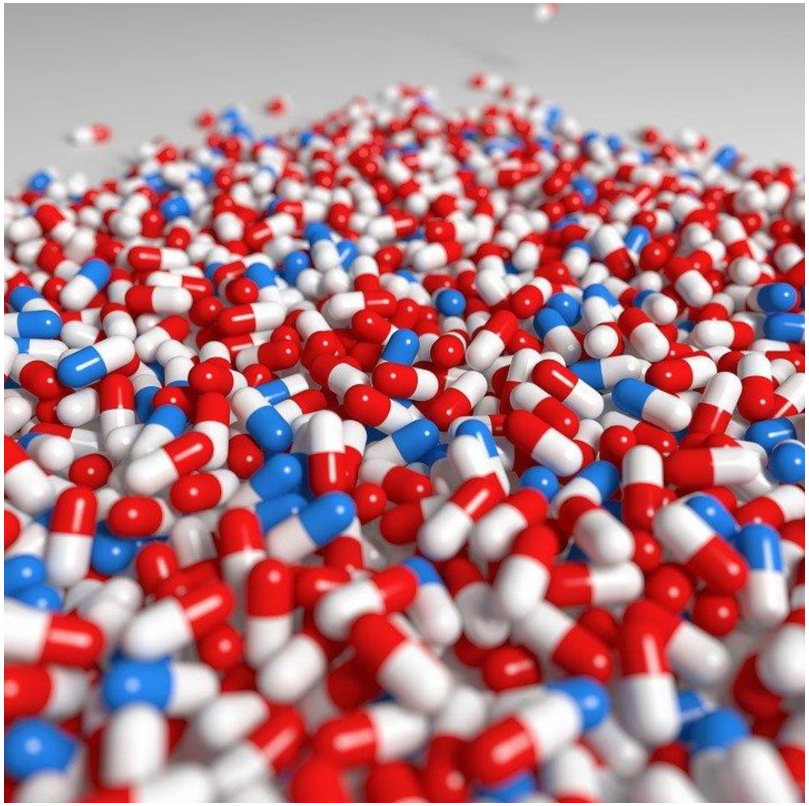

TO MAKE PROXY MEDICINE THAT IS THE SAME IN EVERY WAY!

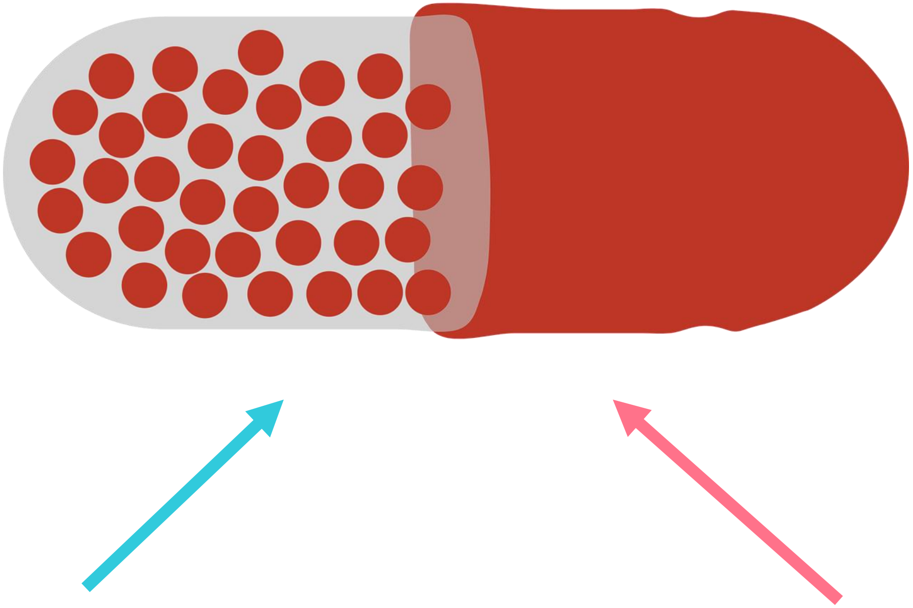

Excipient

80 – 90%

- Cellulose
- Lactose
- Sucrose

Active Pharmaceutical Ingredient (API)

10 – 20%

CAPSULE

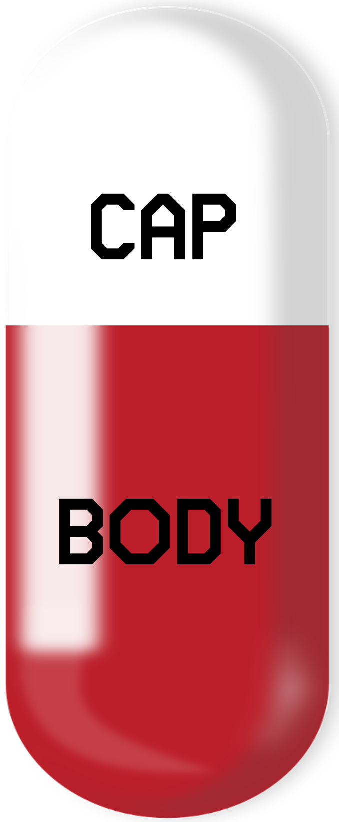

THE GOAL – QUALITY CONTROL

To make medicine that is perfectly formed and exactly the same using a medicine maker kit.

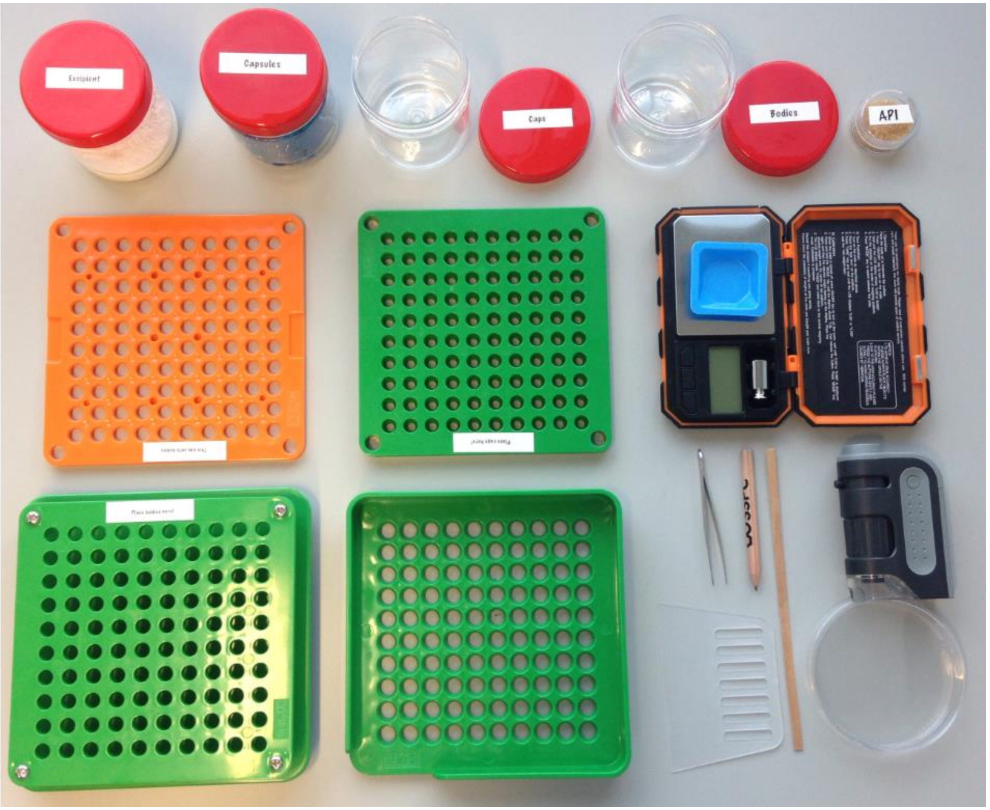

1

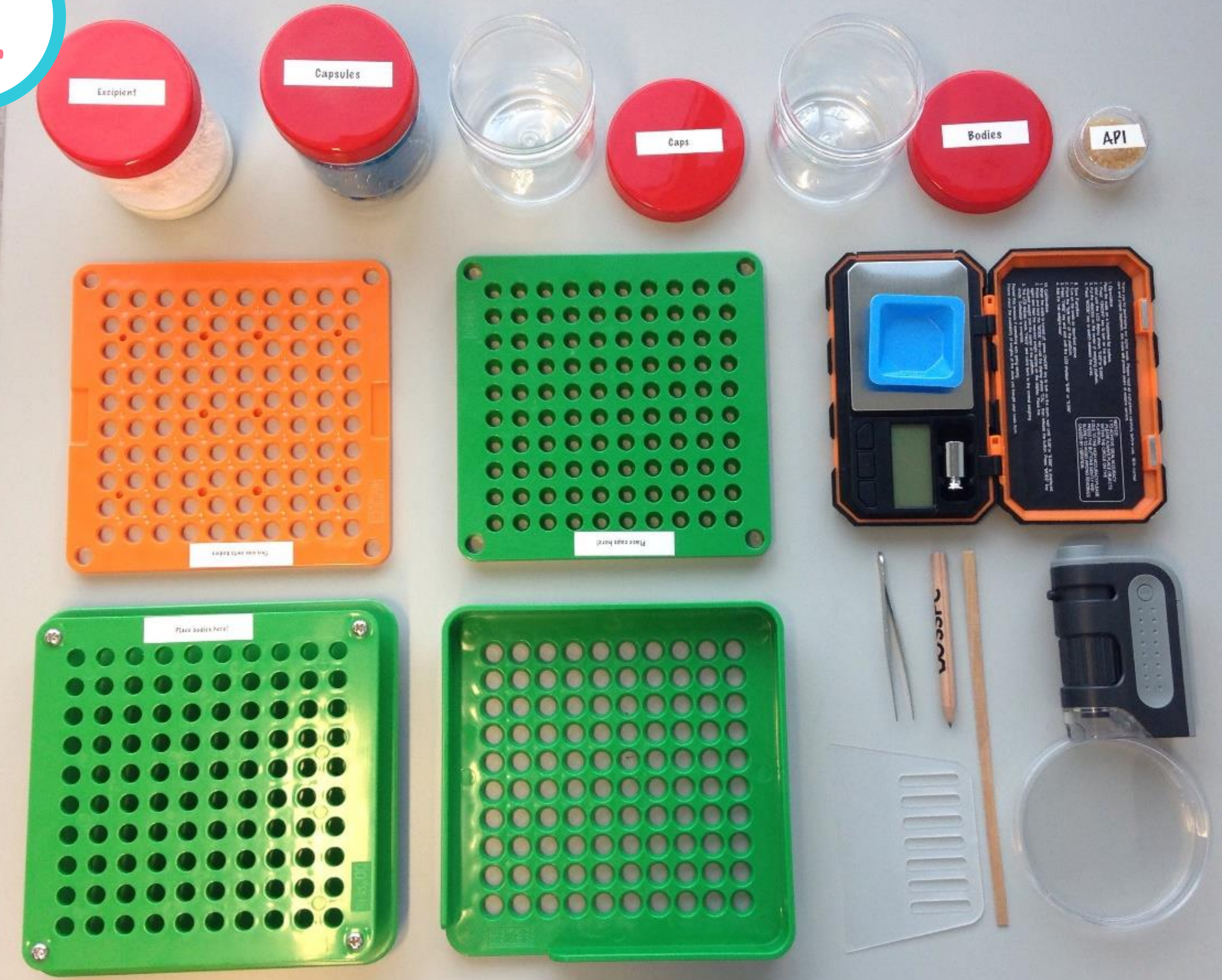

LAY OUT YOUR EQUIPMENT LIKE THIS - 5 MINUTES

2

Get to know the Pill Plate

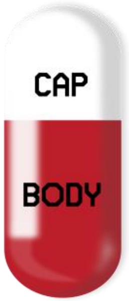

Sorting Plate

Cap Plate

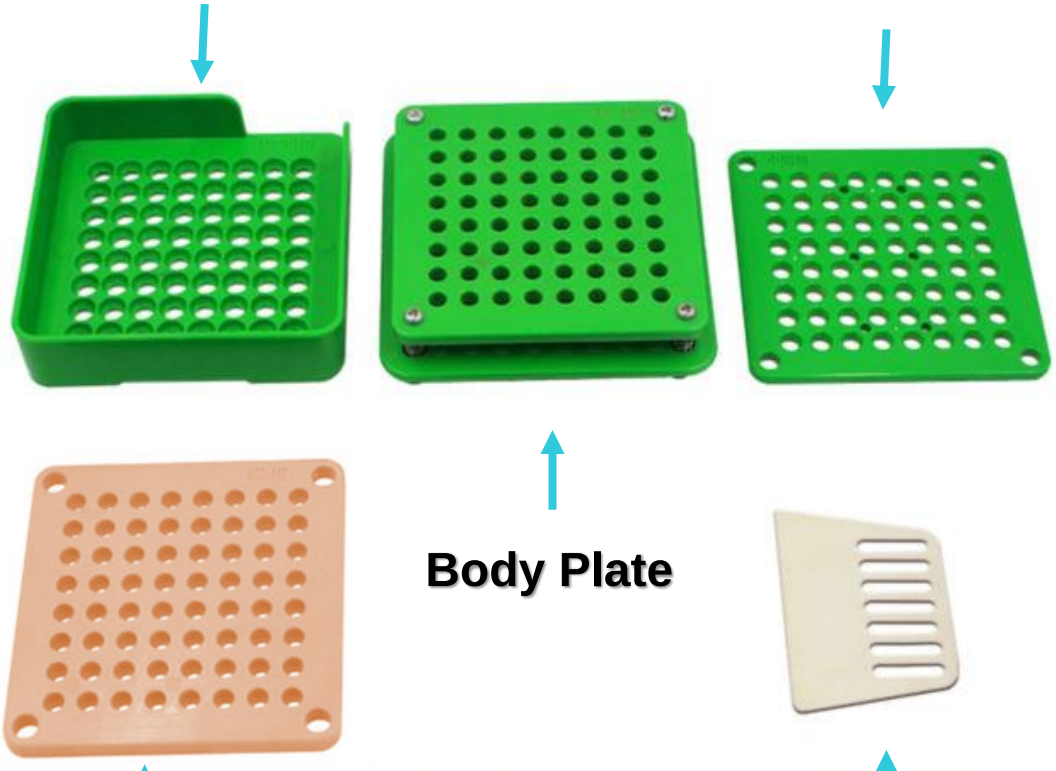

Body Plate

Middle (orange) Plate

Powder Spreader

3

Separate the caps and bodies into the labelled containers with the red lids - 5 mins

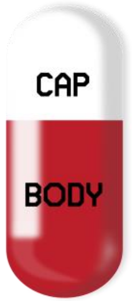

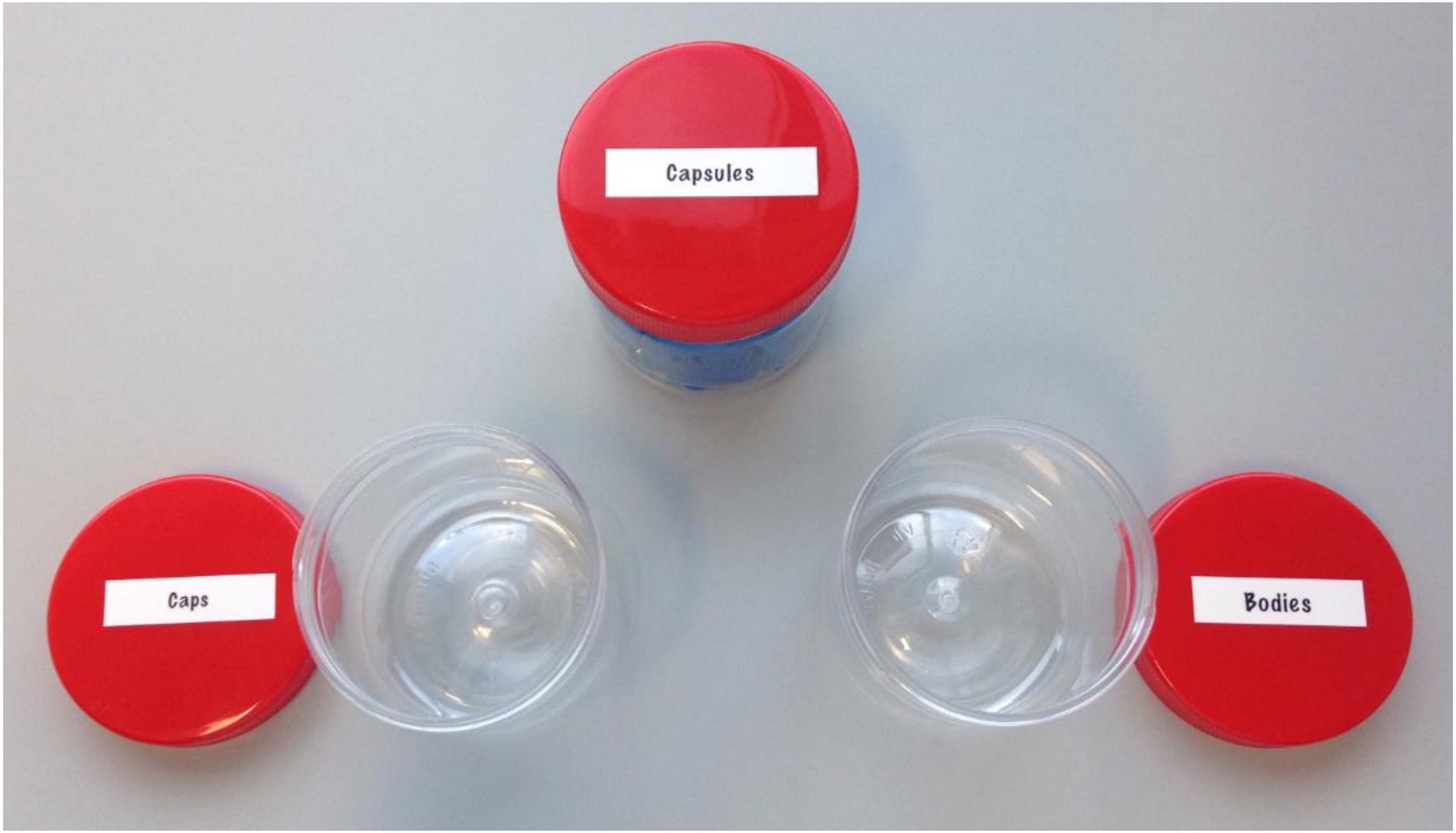

4

Put the sorting plate on the body plate and pour the bodies into the slots

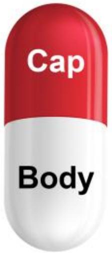

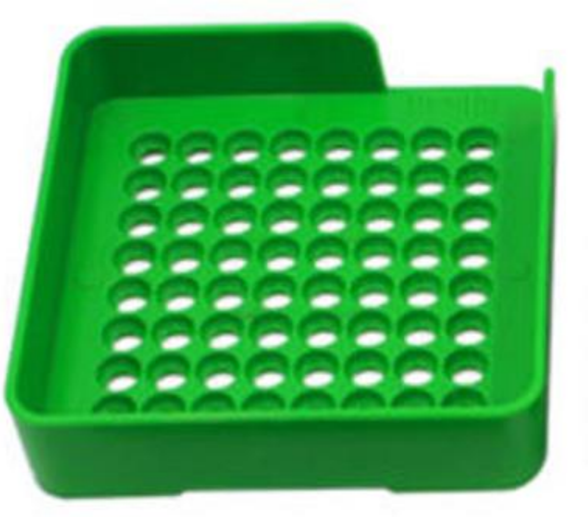

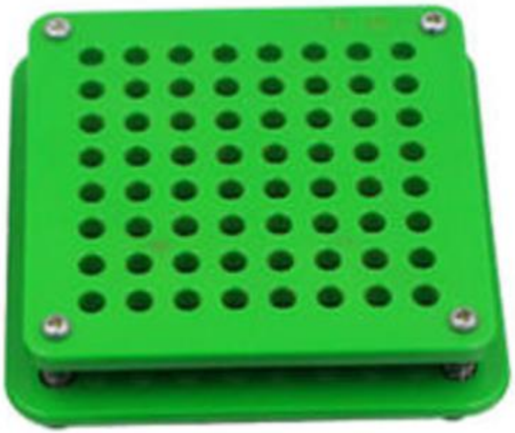

Sorting Plate

Body Plate

5

Fill the pills! Use the API and excipient. Try to make all the pills the same! Do this step inside the lid of the Medicine Maker box.

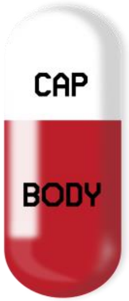

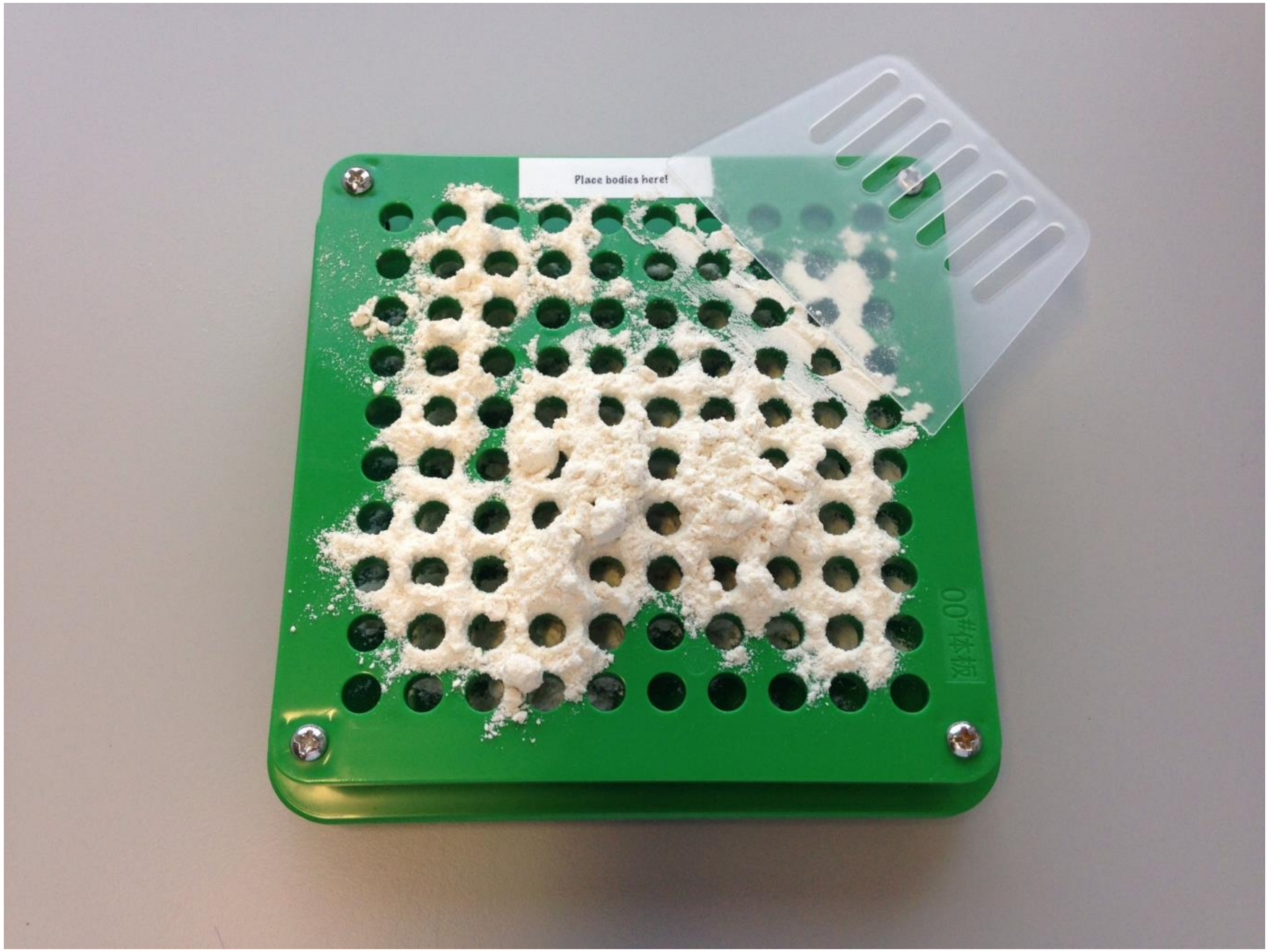

6

Put the sorting plate on the cap plate and pour the caps into the slots.

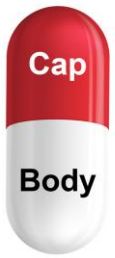

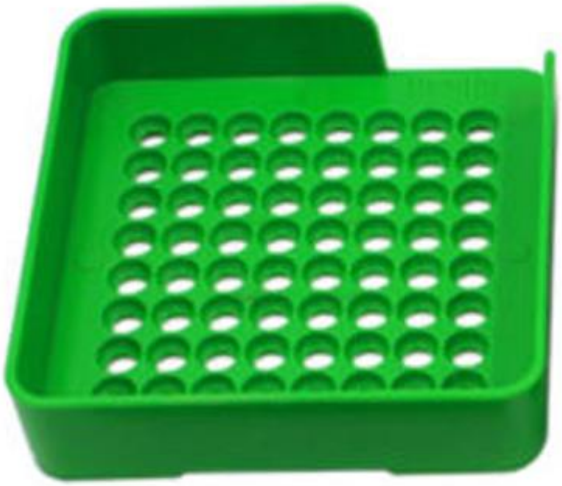

Sorting Plate

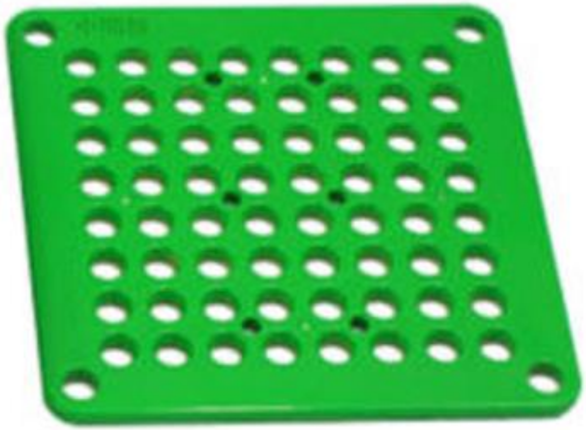

Cap plate

7

Put the middle plate (orange) on the cap plate. Make sure it's the right way around!

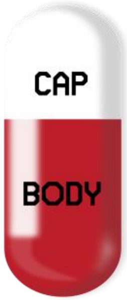

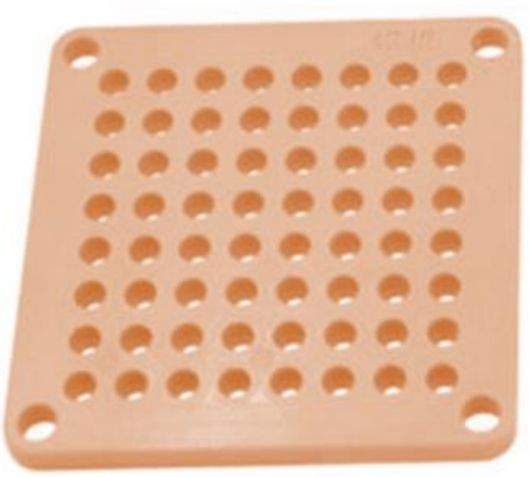

Middle Plate

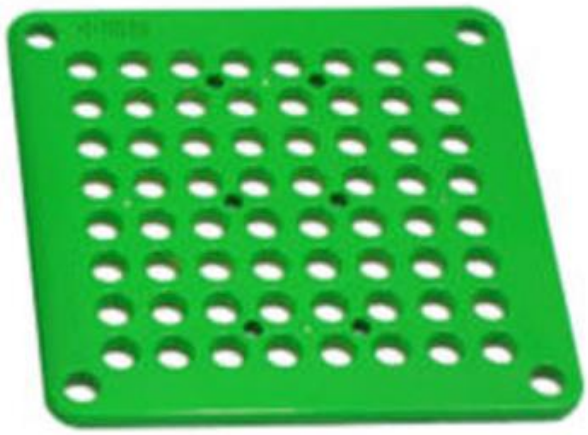

Cap plate

8

Place the middle plate and cap plate onto the body plate in a single motion and press the plates together to connect the caps and bodies.

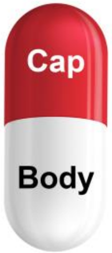

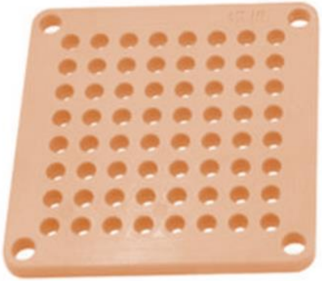

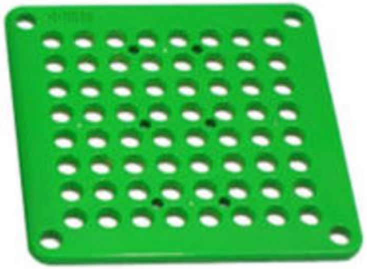

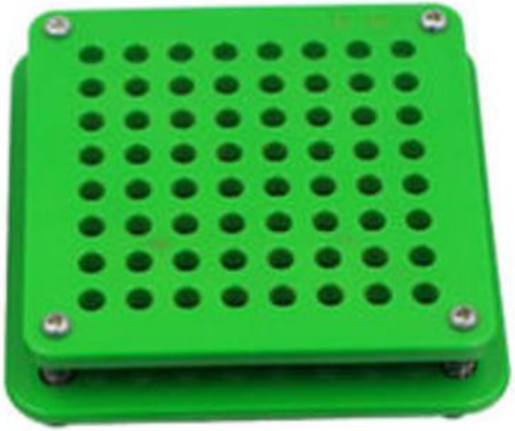

Middle Plate

Cap plate

Body Plate

LET'S CHECK YOUR MEDICINE: 1

Weigh 10 individual pills on the scales and record their weight.

If the lightest and heaviest pill are within 10% of each other, your batch has passed the first test!

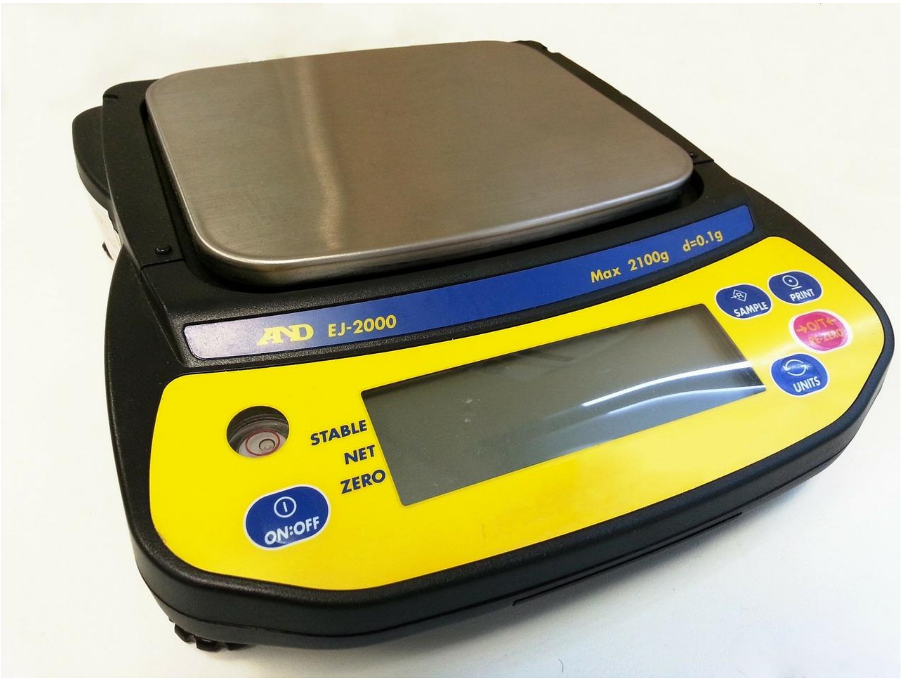

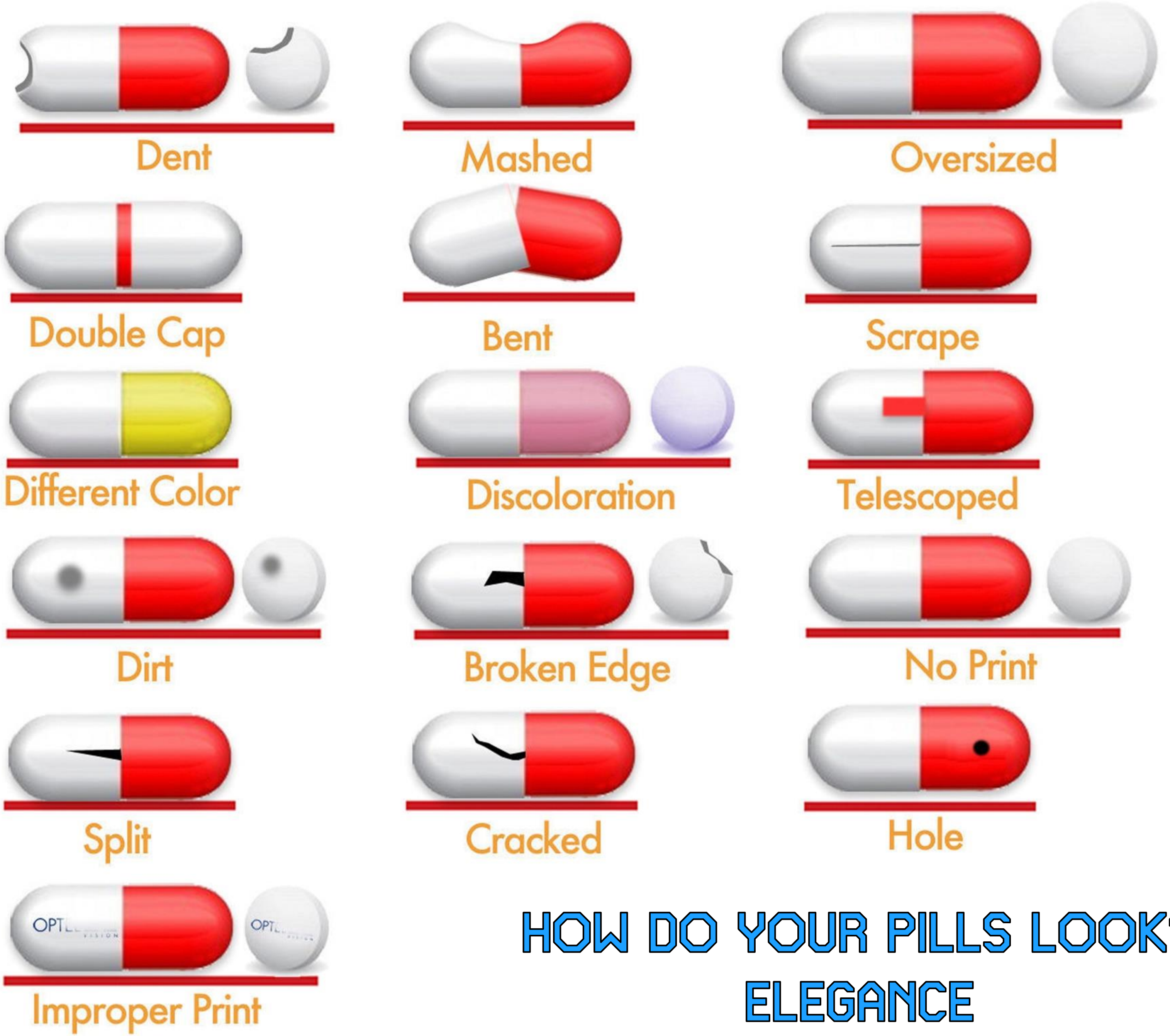

HOW DO YOUR PILLS LOOK?
ELEGANCE

LET'S CHECK YOUR MEDICINE: 2

Empty out two of your pills into the petri dish and see if there is a difference between the content of the pills.

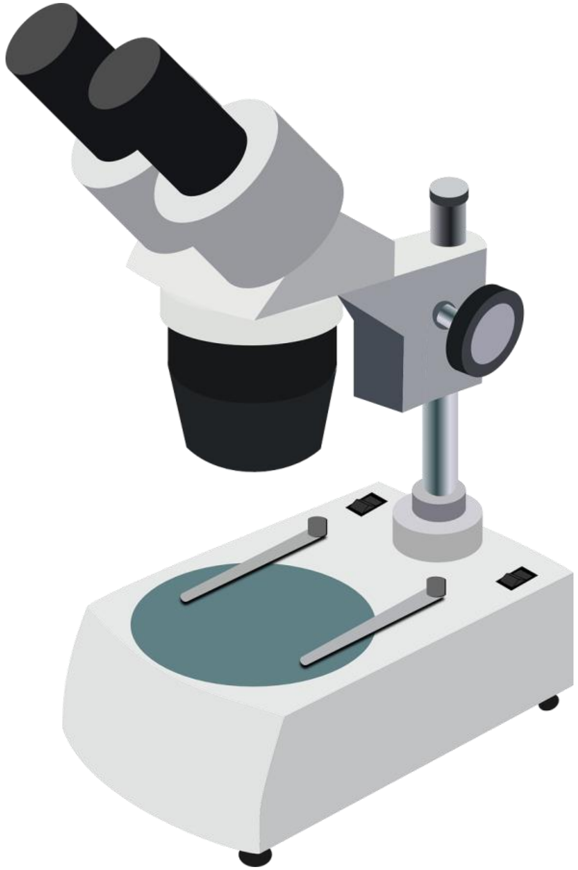

Use the mini microscope to see the difference between the crystal API and the amorphous excipient.

What do you see?

THANK

YOU!

QUESTIONS?

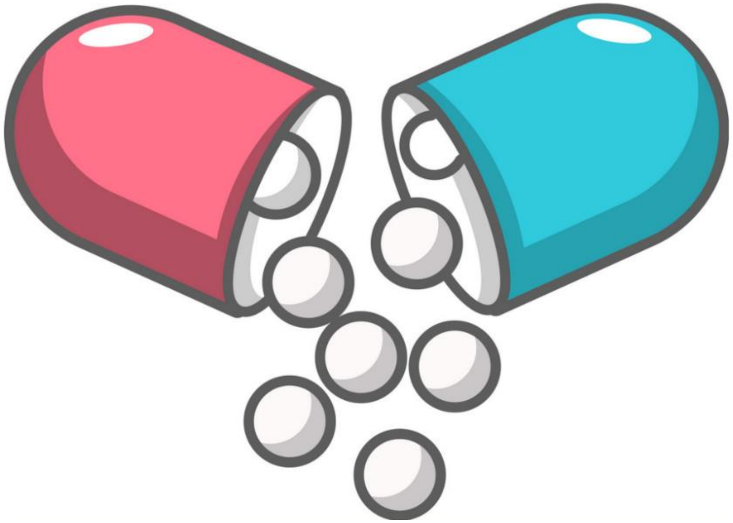

Supplement: Supplementary file 1 — ed1c00915_si_001.pdf [file ed1c00915_si_001.pdf]
